# Predicting topological entanglement entropy in a Rydberg analogue simulator

**DOI:** 10.1038/s41567-025-02944-3

**Published:** 2025-07-28

**Authors:** Linda Mauron, Zakari Denis, Jannes Nys, Giuseppe Carleo

**Affiliations:** 1 Institute of Physics, École Polytechnique Fédérale de Lausanne (EPFL), Lausanne, Switzerland; 2 Center for Quantum Science and Engineering, École Polytechnique Fédérale de Lausanne (EPFL), Lausanne, Switzerland

**Keywords:** Quantum simulation, Topological defects

## Abstract

Predicting the dynamical properties of topological matter is a challenging task, not only in theoretical and experimental settings, but also computationally. Numerical studies are often constrained to studying simplified models and lattices. Here we propose a time-dependent correlated ansatz for the dynamical preparation of a quantum-spin-liquid state on a Rydberg atom simulator. Together with a time-dependent variational Monte Carlo technique, we can faithfully represent the state of the system throughout the entire dynamical preparation protocol. We are able to match not only the physically correct form of the Rydberg atom Hamiltonian but also the relevant lattice topology at system sizes that exceed current experimental capabilities. This approach gives access to global quantities such as the topological entanglement entropy, providing insight into the topological properties of the system. Our results confirm the topological properties of the state during the dynamical preparation protocol, and deepen our understanding of topological entanglement dynamics. We show that, while the simulated state exhibits local properties resembling those of a resonating-valence-bond state, in agreement with experimental observations, it lacks the latter’s characteristic topological entanglement entropy signature irrespective of the degree of adiabaticity of the protocol.

## Abstract (web summary)

A numerical approach capable of simulating large-scale Rydberg atom quantum systems suggests that protocols for preparing topological states can produce experimental signatures of these states without reaching a topological phase.

## Main

In recent years, topological properties of matter have attracted increasing interest^1^, especially following the experimental observation of the quantum Hall effect and chiral spin states. Beyond condensed matter, the introduction of the toric-code model^2^ marked the beginning of a new paradigm in quantum computation known as topological quantum computing^3,4^, which exploits the robustness of topological order to local perturbations to achieve fault-tolerant computations^5^.

Among the various phases of matter exhibiting topological order in two dimensions, quantum spin liquids (QSLs) stand out as particularly intriguing. These states exhibit no magnetic order, strong long-range quantum correlations and anyonic many-body excitations^6^. A paradigmatic model for understanding this phenomenon is the toric code^2^, a lattice ${{\mathbb{Z}}}_{2}$ gauge theory^7,8^. Its fourfold-degenerate ground state includes the so-called vacuum, corresponding to a resonating-valence-bond (RVB) state^6^. Its peculiar anyonic properties make QSLs promising candidates for topologically protected quantum memory^9,10^.

A key feature of topologically ordered systems is their entanglement entropy. In addition to the area-law scaling typical of gapped ground states, these systems exhibit a negative offset, known as the topological entanglement entropy (TEE), which serves as a robust signature of topological order^11^. Characterizing such states remains a challenge for both experiments and simulations. Recent efforts have aimed at their preparation on various platforms^12–16^. In particular, a dynamical preparation protocol for a QSL was demonstrated in Rydberg atom arrays^17^. However, unambiguous detection of topological order remains difficult due to the necessity for global probes. Entanglement-based signatures are especially hard to access experimentally, and the scalability of quantum-state tomography^18^, replicas^19^ and other techniques^20,21^ is still under active investigation. As a result, experiments often rely on classical numerical simulations.

From a numerical standpoint, simulating quantum dynamics remains an important challenge. Standard approaches such as tensor networks^22^ suffer from entanglement growth during time evolution^23^, which demands increasing computational resources and restricts simulations to small systems and specific geometries and boundary conditions. This is especially restrictive for density matrix renormalization group methods^24,25^, typically limited to narrow cylinders. Additional complexity arises when matching experimental settings, such as long-range interactions present in Rydberg atom lattices^26^.

Developing accurate and efficient simulation techniques is therefore crucial, and variational Monte Carlo (VMC) offers an appealing alternative. From simple mean-field ansätze to deep neural quantum states^27^, it offers versatility in the structure of the variational wave function. VMC lifts the restriction on entanglement and allows full control over system geometry, boundary conditions and topology (for example, genus), which is essential for studying topological states. The time-dependent variational Monte Carlo (t-VMC) algorithm^28–30^ further enables scalable simulation of unitary evolution, which makes it suitable for modelling experimental state preparation protocols.

In this work, we demonstrate that topologically ordered phases can be efficiently simulated using t-VMC, while retaining fine control over the system geometry. This method provides direct access to the time-dependent wave function, allowing for measurement of various quantities, including relevant observables and the TEE. We focus on the dynamical preparation of a QSL using a Rydberg atom simulator. Our approach captures key experimental features, from the long-range van der Waals interactions intrinsic to Rydberg platforms to the lattice geometry and boundary conditions. We show that a lightweight variational ansatz, with a number of parameters scaling linearly with system size, accurately represents both the ideal QSL and the full dynamical evolution. This enables simulations to reach up to 288 atoms, beyond the lattice sizes reported in experiments^17^. Although our numerical results are consistent with the observables measured in experiments, we can directly probe the dynamics of the topological entanglement, thereby discussing the presence of topological order at the end of the protocol.

## Topological entanglement entropy

The entanglement between a region A and its complement $\bar{\mathrm{A}}$ may be quantified by the entropy *S* of its reduced density matrix${\hat{\rho }}_{\mathrm{A}}={{\rm{Tr}}}_{\bar{\mathrm{A}}} \left\vert \psi \right\rangle \left\langle \psi \right\vert$. Although this quantity follows an area-law scaling for the ground state of any gapped Hamiltonian^31^, topologically ordered states present the following crucial correction:

1 $$S({\hat{\rho}}_{\mathrm{A}})=\alpha L-\gamma +{\mathcal{O}}(1),$$

where *L* is the length of the boundary between the two regions A and $\bar{\mathrm{A}}$, *α* is a non-universal factor and the offset −*γ* < 0 is the TEE, a fundamental feature of such states. The term ${\mathcal{O}}(1)$ vanishes in the thermodynamical limit.

The area-law behaviour (equation (1)) serves as evidence of the local entanglement near the boundaries of the two regions, while the value of the TEE provides a direct indicator of long-range entanglement and topological order. The TEE directly characterizes the type of topological order. The anyonic properties define the total quantum dimension ${\mathcal{D}}=\sqrt{{\sum }_{a}{d}_{a}^{2}}$, where *d*_*a*_ denotes the local dimension of particles within superselection sector *a*^32,33^. The corresponding TEE then reads $\gamma =\ln ({\mathcal{D}})$. The present study focuses on characterizing a QSL with ${{\mathbb{Z}}}_{2}$ topological order, thus characterized by $\gamma =\ln (\sqrt{4})=\ln (2)$.

## Rydberg quantum simulator

**Fig. 1:**
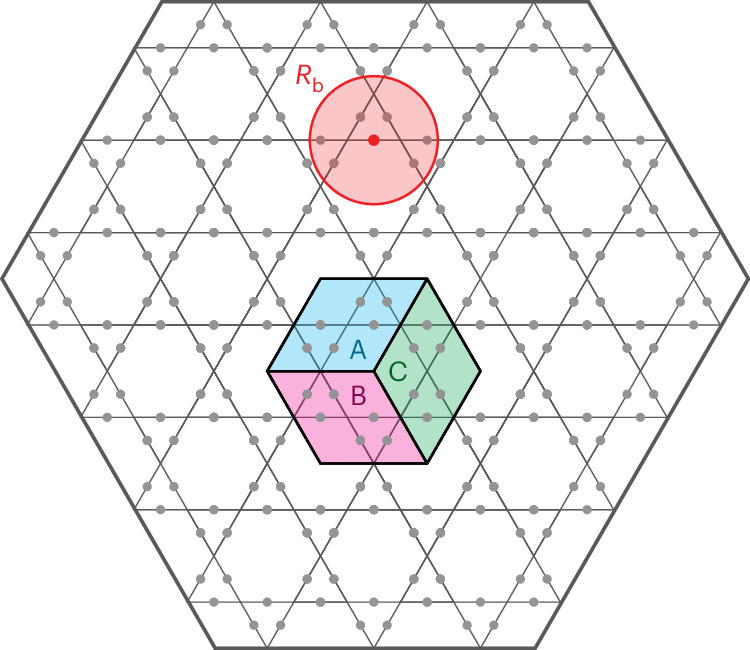
Kagome lattice used in the experiment. The red circle represents the blockade radius *R*_b_ = 2.4*a* set during the process. Subsets A, B and C indicate the tripartition considered to estimate the TEE (equation (10)) throughout all this work.

We consider a two-dimensional array of Rydberg atoms placed at the edges of a Kagome lattice, as introduced in ref. ^17^ and depicted in Fig. 1. Each atom can either be in its electronic ground state |*g*〉 or excited to |*r*〉 via an optical transition with the Rabi frequency *Ω*_0_. Excited Rydberg atoms interact via a repulsive van der Waals potential *V*(*r*) = *V*_0_/*r*^6^ leading to the Rydberg blockade, a phenomenon whereby atoms within a characteristic distance *R*_b_ = (*V*_0_/*Ω*_0_)^1/6^ cannot be simultaneously excited. The Hamiltonian for such a system is given by

2 $$\hat{{\mathcal{H}}}(t)=-\frac{\varOmega (t)}{2}\sum _{i}{\hat{\sigma }}_{i}^{\;x}-\varDelta (t)\sum _{i}{\hat{n}}_{i}+\sum _{i < j}V({r}_{ij}){\hat{n}}_{i}{\hat{n}}_{j},$$

where ${\hat{\sigma }}_{i}^{\;x}= \left\vert {r}_{i}\right\rangle \left\langle {g}_{i}\right\vert +\left\vert {g}_{i}\right\rangle \left\langle {r}_{i}\right\vert$ and ${\hat{n}}_{i}= \left\vert {r}_{i} \right\rangle \left\langle {r}_{i} \right\vert$. Here the Rabi frequency *Ω*(*t*) governs the strength of the coherent driving of the Rydberg transition whereas the time-dependent detuning *Δ*(*t*) serves as a chemical potential controlling the number of excitations in the system.

We set the lattice spacing *a* = 3.9 μm and *Ω*_0_ = 2π × 1.4 MHz, yielding *R*_b_ = 2.4*a*, that is a blockade extending to third-nearest neighbours (Fig. 1). On a Kagome lattice, this implies that at most one atom per triangle and per vertex is likely to be excited, effectively realizing a dimer model. Following ref. ^34^, ground and excited states are mapped to the absence and presence of dimers, |*g*〉 = |—〉 and , respectively; for example,  on a triangle.

### Dynamical state preparation

Recent studies^34–41^ have shown that truncated Rydberg Hamiltonians exhibit a QSL ground state for certain values of *Δ*. With exactly one dimer per vertex, the ground state is a coherent superposition of compact dimer coverings, akin to the RVB state^6^. However, these simplified models ignore the long-range character of *V*(*r*), considering only up to fourth-nearest-neighbour interactions. Numerical studies^42^ have revealed that the neglected long-range tails preclude the existence of a QSL ground state, favouring instead a valence-bond solid. Interestingly, dynamical state preparation appears more robust, yielding QSL-like phases despite the full interaction potential.

**Extended Data Fig. 1:**
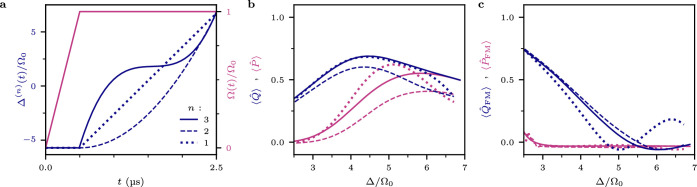
Comparison of different protocols through exact evolution of a small lattice of *N* = 24 atoms. **a** Time dependence of the parameters of the Hamiltonian for all considered protocols. All start with *Ω*(0) = 0 and *Δ*(0) = − 2*π* × 8 MHz and take place over a total time *T* = 2.5 *μ*s. The Rabi frequency *Ω*(*t*) is first linearly increased up to the target value *Ω*_0_ = 2*π* × 1.4 MHz during *t* = *T*/5, while the detuning *Δ*(*t*) is kept constant. The detuning is then ramped up following a polynomial of degree *n* up to its final value *Δ*(*T*) = 2*π* × 9.4 MHz. The protocol in the main text uses *n* = 3 and corresponds to that of ref. ^17^. **b** Expectation value of the topological string operators $\hat{Q}$ and $\hat{P}$ for the various forms of *Δ*(*t*) upon time evolution. **c** Expectation value of ${\hat{Q}}_{{\rm{FM}}}$ and ${\hat{P}}_{{\rm{FM}}}$ for the same simulations. Source data

A dynamical protocol compatible with Rydberg platforms was proposed in ref. ^17^ to prepare and probe a QSL. The system is initialized in the state |*ψ*(*t* = 0)〉 = |*g*〉^⊗*N*^, the unique ground state for *Ω*(0) = 0 and *Δ*(0) < 0. The state then evolves under the time-dependent Hamiltonian in equation (2), with both *Ω*(*t*) and *Δ*(*t*) ramped up (see Extended Data Fig. 1 and Supplementary Section 1 for details), eventually reaching *Δ* > 0, where the Rydberg density satisfies $\langle \hat{n}\rangle \approx 1/4$. To reach the QSL metastable phase, the evolution must be faster than adiabatic to avoid remaining in the topologically trivial instantaneous ground state, yet slow enough to suppress excitations to higher-energy states.

### String operators

Since the entanglement entropy cannot be accessed experimentally at relevant system sizes, alternative probes are needed to identify the QSL phase. Given the link between anyonic properties and topological order, following ref. ^34^ we define topological string operators in the dimer representation34In these equations, h.c. stands for Hermitian conjugate. The non-diagonal operator $\hat{Q}$ acts on wiggly strings  by shuffling the dimers on crossed triangles. When applied on a closed contour, ${\hat{Q}}^{{\rm{hex}}}$ maps every valid dimerized configuration to another valid dimerization, probing their coherence. The diagonal operator $\hat{P}$ acts on edges crossed by the dashed line  and its parity on a single dimerization signals defects. Thus, ${\hat{P}}^{{\rm{hex}}}$ quantifies the quality of the dimerizations present in the state. In the ${{\mathbb{Z}}}_{2}$ vacuum, both ${\hat{Q}}^{{\rm{hex}}}$ and ${\hat{P}}^{{\rm{hex}}}$ evaluate to 1. For clarity, we omit superscripts hereafter.

When defined on an open contour, either operator acting on the vacuum state results in the creation of anyons at both ends, thereby generating an (orthogonal) excited state. This leads to the Fredenhagen–Marcu (FM) order parameters:5These vanish for a QSL and thus constitute a good probe, although they can become ill defined outside this phase.

## Dynamics of the topological operators

**Fig. 2:**
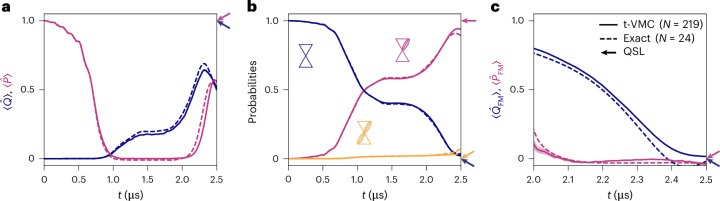
Simulation of the dynamical state preparation. **a**–**c**, Variational results (t-VMC) on a ruby lattice of *N* = 219 atoms (as introduced in Fig. 1). Solid lines indicate the Monte Carlo averages during t-VMC. Dashed lines show the exact simulation for a small lattice of *N* = 24 atoms (as defined in Methods). Side arrows denote the expected values for an ideal QSL. Evolution of the $\hat{P}$ (purple) and $\hat{Q}$ (dark blue) operators, as defined in equations (3) and (4) (**a**), the probabilities to find a monomer (dark blue), dimer (purple) or double dimer (yellow) in the evolved state (**b**), and the FM order parameters, as defined in equation (5) (**c**). The FM order parameters are only computed at large times since their definition is only meaningful where the closed loops take finite values. All observables are averaged over all instances (vertices, hexagons, half-hexagons) present in the bulk of the lattice. Statistical confidence intervals are narrower than the line width for all observables. Source data

**Extended Data Fig. 2:**
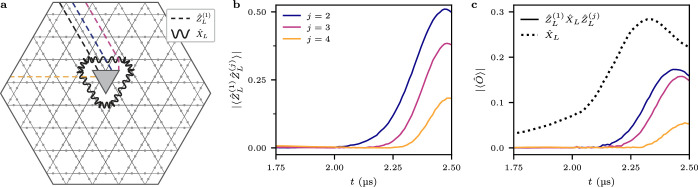
Definition and measurement of logical operators. **a** Schematic illustration of the pierced lattice with *N* = 285 Rydberg atoms and definition of the logical operators. The filled gray triangle represents a hole in the lattice where no atom is present, and therefore acts as an additional boundary. The logical operator ${\hat{X}}_{L}$ (black wiggly line) acts on a string looping around the central hole and ${\hat{Z}}_{L}$ (dashed lines) acts on sites connecting the hole to the external boundary. The action of both operators on the logical states is described in Supplementary Equation (1). We define multiple operators ${\hat{Z}}_{L}^{(j)}$ corresponding to various choices of topologically equivalent paths. **b** Measurements of the Pauli identity $\hat{{\mathbb{1}}}={\hat{Z}}_{L}\times {\hat{Z}}_{L}$ for various pairs of logical operators. As the sign of the expectation value depends on the number of enclosed vertices, the absolute value is plotted here. **c** Verification of the identity ${\hat{X}}_{L}=-{\hat{Z}}_{L}\times {\hat{X}}_{L}\times {\hat{Z}}_{L}$ for our logical operators. As for **b**, we take the absolute value due to the dependence of the sign on the number of enclosed vertices. Source data

Since all relevant observables are intensive, we can directly compare results across system sizes. Figure 2 shows variational results for the full lattice (*N* = 219) alongside exact dynamics for a smaller system (*N* = 24). The onset of a QSL phase occurs around *t* = 2.0 μs, marked by a shift in all observables toward their expected QSL values. However, the topological operators never fully match their ideal predictions. The presence of residual monomers and occasional double dimers suppresses $\hat{P}$, while $\hat{Q} < 1$ reflects that the state is not an equal-weight superposition of dimer configurations, as in a true QSL. Moreover, a detailed study of the effect of the topological operators on the topologically protected manifold reveals a substantial dependence on the distance between the operators, even for topologically equivalent operators, highlighting the locality of the QSL-like features (Extended Data Fig. 2 and Supplementary Section 2).

While the FM order parameters begin converging alongside other observables, both ultimately reach the expected QSL value of zero. $\langle {\hat{P}}_{{\rm{FM}}}\rangle$ is zero valued throughout the whole dimerized regime, *t* > 2.0 μs, offering little insight into the precise location of the QSL phase. In contrast, the off-diagonal order parameter drops to $\langle {\hat{Q}}_{{\rm{FM}}}\rangle \approx 0$ at *t* ≳ 2.4 μs, suggesting a QSL regime for *Δ*/*Ω*_0_ ≳ 5.5. Although this supports the emergence of a QSL, the important discrepancy observed for the topological operator underscores the limitations of FM order parameters in detecting deviations from an ideal QSL.

**Extended Data Fig. 3:**
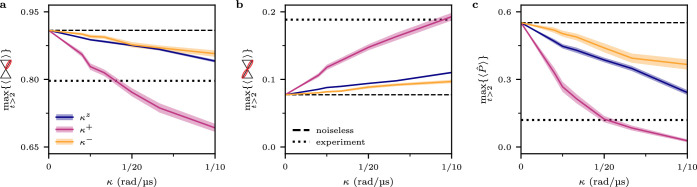
Effect of noise channels on observables. Each color corresponds to the simulation of the protocol subject to solely one noise channel, as defined in Supplementary equation (4), with increasing rate *κ*^*α*^ in the absence of spatial disorder. Noisy simulations (solid lines) are carried out on the smaller lattice (*N* = 24) defined in Methods. The results are compared to the noiseless exact evolution on the same lattice (dashed) and to the experimental results from ref. ^17^ obtained on the real-scale lattice in Fig. 1 (dotted). Data is obtained as the mean value over 100 quantum trajectories and lighter bands indicate the root mean square error. **a** Maximal probability to find dimers on the vertices of the lattice. **b** Maximal probability to find double dimers. **c** Maximal value of the diagonal operator $\langle \hat{P}\rangle$. All plotted quantities correspond to the maximal values inside the QSL-like phase for *t* > 2.0 *μ*s. Source data

**Extended Data Fig. 4:**
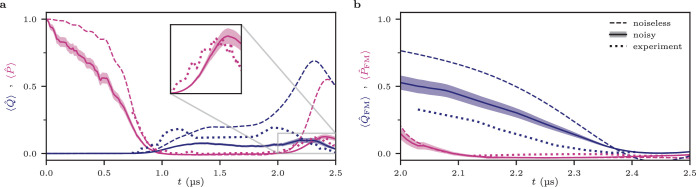
Effect of noise on the dynamics. Effect on **a** the topological string operators and **b** FM order parameters for a small lattice of *N* = 24 Rydberg atoms. The different colors indicate the measured operators. Different line styles denote different settings: noiseless exact evolution (dashed), noisy evolution (solid) and experimental measurements from ref. ^17^ on a lattice of *N* = 219 atoms (dotted). The results obtained for $\langle \hat{P}\rangle$ are highlighted in the inset. The statistical confidence intervals (RMS error over 100 quantum trajectories) are represented by lighter bands. The details of the choice of the noise parameters are discussed in Supplementary Section S3.2. Source data

We further verify that, upon considering non-idealities, our results match those of the experiment (see Extended Data Figs. 3 and 4 and Supplementary Section 3 for further details). Moreover, this allows us to quantify experimental errors associated with the measurement of off-diagonal operators.

## Dynamics of topological entanglement entropy

**Fig. 3:**
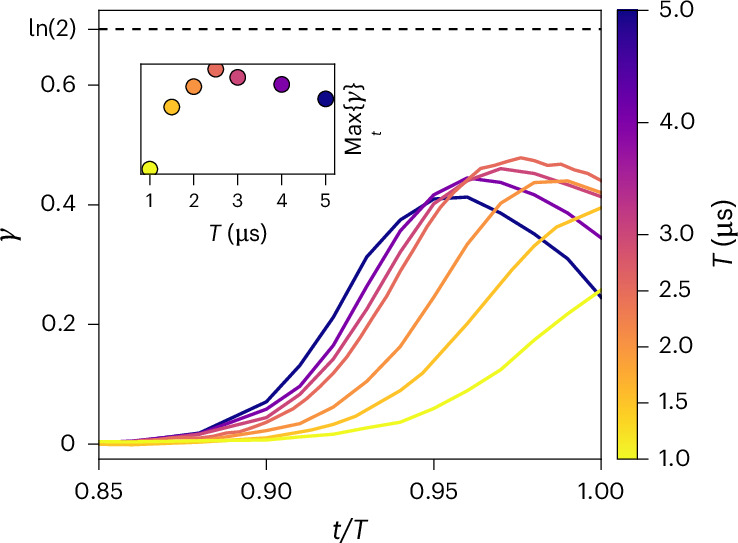
Dynamics of the TEE for various total preparation times. Solid lines show the results obtained during time evolution for each protocol, denoted by a different-coloured line. The values and corresponding errors are obtained from 1,024 bootstrap estimates. Statistical confidence intervals are narrower than the line width. The dotted line indicates the value obtained for the RVB form of the state (equation (8)), corresponding to *γ* = ln(2). The inset displays the highest *γ* reached for each simulation. The simulation with total time *T* = 2.5 μs corresponds to the experimental protocol from ref. ^17^. Source data

To further characterize the long-range entanglement of the dynamically prepared state, we estimate the TEE in the same setting, using the Kitaev–Preskill prescription on the tripartition defined in Fig. 1 (Methods). As shown in Fig. 3, the TEE remains negligible up to *t* = 0.9*T* = 2.25 μs, consistent with the absence of topological order in the trivial phase at low *Δ*. At later times, a finite TEE is observed, peaking at *γ* ≈ 0.479(2) around *t* ≈ 2.44 μs (*Δ*/*Ω*_0_ ≈ 6). However, the system never reaches the characteristic value *γ* = ln(2) for ${{\mathbb{Z}}}_{2}$ order, indicating that the final state is not a pure ${{\mathbb{Z}}}_{2}$ QSL.

**Extended Data Fig. 5:**
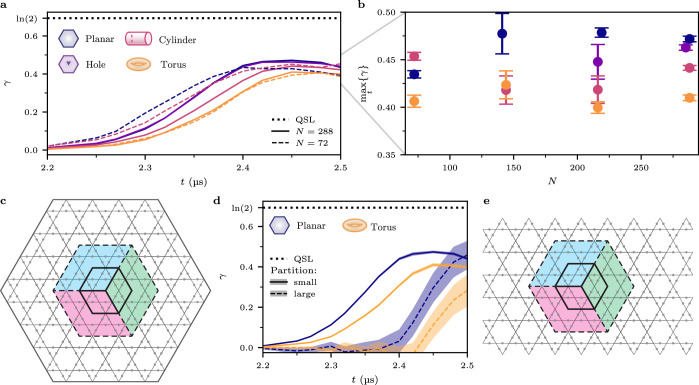
Topological entanglement entropy for various geometries. **a** TEE throughout time evolution for the smallest (*N* = 72) and biggest (*N* = 288) systems of each lattice geometry. Geometries are the main lattice with open boundary conditions as presented in Fig. 1 (Planar), the same lattice with a hole in the middle (Hole), a cylindrical (Cylinder) and a toric one (Torus), with respectively one and two periodic boundaries. The TEE is calculated for the partition depicted in Fig. 1, optimally centered in the bulk of each lattice. **b** Maximal value of the TEE for each geometries at various system sizes. The planar lattice with a hole was not simulated for a lower number of particles since such a lattice would not possess any bulk. **c** and **e** Illustration of the biggest lattices, with zero (Planar) and two (Torus) periodic boundary conditions respectively. Two different tri-partitions (small and large) are illustrated on both lattices. **d** TEE throughout time evolution for the systems presented in **c** and **e**. Solid lines stand for the small tri-partition and dashed the large one. The dotted line indicates the value obtained for an RVB state in both cases. The color of the lines indicate the lattice simulated, as encoded in the top panels. The values and statistical confidence interval were obtained from 1,024 bootstrap estimates. Source data

Unlike previous works focused on cylindrical or toroidal geometries, our set-up involves open boundaries, as relevant for experiments. Since the topology of the lattice can ultimately affect the final state, we study its influence on the TEE in Supplementary Section 4 (Extended Data Fig. 5). This analysis confirms that variations in boundary conditions, genus or system size do not account substantially for the reduced TEE. Notably, none of the alternative lattice configurations led to an improvement in *γ*. Additionally, in Supplementary Section 4, we study the influence of the size of tripartition used in the Kitaev–Preskill prescription and observe that the reduced value of *γ* cannot be compensated for by expanding the size of the domains.

As previously discussed, the preparation protocol with long-range Rydberg interactions must strike a sensitive balance between adiabaticity and destabilization (due to the tails) to reach the QSL phase. Using the TEE as a global marker of topological order, we study its dependence on the protocol duration *T* in Fig. 3. We consistently observe a subideal TEE for *t* ≳ 0.9*T*, with the peak shifting earlier for longer *T* and later for shorter *T*.

Figure 3 also reports the maximum value of *γ* across different preparation times *T*, with the optimal value *γ* ≈ 0.479(2) occurring for *T* = 2.5 μs, as used in ref. ^17^. As *γ* remains below ln(2) for all *T*, we conclude that the prepared state lacks the signature of pure ${{\mathbb{Z}}}_{2}$ topological order, and thus does not correspond to an ideal RVB state.

## Perturbative effects

Our results show reduced values across all indicators of topological order. However, finite-size effects and local perturbations may reduce these quantities while not causing the system to exit the topologically ordered phase^7^. In particular, the presence of a spurious transverse field may drive the system toward a trivial magnetic phase, but might also, for moderate strengths, only impact observables without destroying the long-range topological order. To discard this possibility, we study its effect on the ground state of the toric-code Hamiltonian. Here, this corresponds to a PXP model acting on the dimers of the Kagome lattice:6

The effect of this perturbation can be better understood through the anyonic properties of the topological state. While deep in the topological phase anyons can only be created in pairs, the perturbation allows anyons to spontaneously appear, move and interact. The presence of such dynamic anyons can directly impact the value of observables, such as topological operators and TEE, even though the state still possesses a ${{\mathbb{Z}}}_{2}$ topological order. We thus want to elucidate whether the reduction of all signatures reflects genuine loss of topological order or stems from dynamical-matter effects introduced by the long-range tails.

**Fig. 4:**
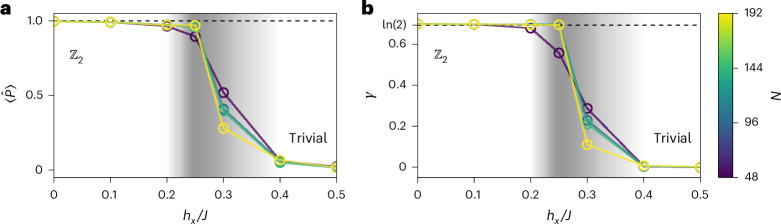
Phase transition of the perturbed toric-code model. **a**,**b**, Each circle denotes the measurement performed on the ground state of the Hamiltonian (equation (6)) for increasing values of *h*_*x*_ using VMC. All systems possess both periodic boundary conditions and were optimized following the procedure described in Methods. The colours stand for the size of the system simulated. **a**, Monte Carlo average of the topological operator $\hat{P}$ acting on a closed hexagon. **b**, Bootstrap estimate of TEE with 1,024 bootstraps, using the same prescription as for other systems in this work. Statistical confidence intervals are narrower than the marker size. Source data

We present in Fig. 4 the ground-state study of this perturbed Hamiltonian for increasing values of the transverse field *h*_*x*_ at constant coupling *J = 1* on lattices of varying size with periodic boundary conditions. In Fig. 4a, the expectation value of the diagonal plaquette operator  shows a sharp transition from the topologically ordered regime ($\langle \hat{P}\rangle =1$ for *h*_*x*_/*J* ≈ 0) to the trivial magnetic phase ($\langle \hat{P}\rangle =0$ for *h*_*x*_/*J* ≫ 0). We observe no finite-size effect within the topological phase, and moderate effects in the magnetic phase close to the critical point. Notably, convergence to the thermodynamic limit appears already at *N* = 48, well below the system sizes used in our dynamical simulations. This rules out finite-size effects, particularly on the torus, as further discussed in Supplementary Section 4.

For a complete analysis, we further present in Fig. 4b the TEE across the phase transition. Remarkably, the TEE exactly matches the ideal value *γ* = ln(2) within the entire topological phase, even at finite *h*_*x*_. Around *h*_*x*_/*J* = 0.25, *γ* abruptly drops to zero, signalling the transition to a topologically trivial phase. A precise critical point for the phase transition can thus be identified. This analysis highlights the robustness of *γ* against local perturbations (transverse field) and confirms its reliability as an indicator of topological order in this context.

Finally, for systems larger than *N* = 48, finite-size effects in *γ* and $\hat{P}$ are only present within the trivial phase. Similarly, a reduced value of *γ* is only observed outside the topologically non-trivial region of the phase diagram. This indicates that the reduction of the signatures observed during the dynamical protocol is incompatible with both mere finite-size effects and spurious local transformations of an ideal RVB state.

## Conclusions and outlook

In this work, we propose time-dependent VMC as a method to simulate quantum many-body systems with topological order. Unlike previous numerical methods, our approach tracks the full dynamical protocol with the complete first-principles Hamiltonian, without entanglement-growth or interaction-range constraints, and supports arbitrary geometry and topology. Its low number of parameters and flexibility allow experimentally relevant lattice sizes, providing direct access to observables and entanglement measures.

We apply this to the dynamical preparation of a QSL on interacting Rydberg atom arrays. Our method captures the QSL onset and represents the system at all stages. We simulate lattices up to 288 atoms with various boundary conditions and genus 0 and 1 topologies. We compute topological operators, FM order parameters and time-resolved TEE. Although some observables hint at QSL-like properties, the TEE deviates substantially from the ideal value for an RVB state, ruling out true ${{\mathbb{Z}}}_{2}$ order.

Optimizing experimental protocols via simulation is increasingly important^36,43,44^, and we expect our variational approach, which handles long-range interactions and realistic geometries, to become a leading tool. A further application is variational quantum state tomography^45^, enabling estimation of entanglement and other quantities from measurement data. Finally, our methods naturally extend to more expressive wave functions, including neural quantum states, aligning with recent advances^46–49^.

## Methods

### Hilbert space

Considering a system of *N* spins 1/2 whose Hilbert space is spanned by {|↑〉, |↓〉}^⊗*N*^, we map the dimer representation to this space using the correspondence $\left\vert \uparrow \right\rangle =\left\vert g\right\rangle$ = |—〉 and .

The Rydberg blockade is strongest for first-neighbour interactions. Indeed, for distances smaller than *R*_b_, the potential takes values ,  and . Since the wave function of states close to the ground state is expected to have vanishing amplitudes for such configurations due to their high-energy contributions, we effectively work in a constrained space , as introduced in ref. ^34^. The usage of a total first-nearest-neighbour blockade has the advantage of reducing the number of states of the considered Hilbert space, as well as discarding high-frequency terms in the Hamiltonian, adding stability to the whole numerical scheme. Moreover, we verify in Supplementary Section 3 that simulations in this restricted Hilbert space faithfully describe the experimental set-up, even in the presence of non-idealities.

### Variational wave function

Since the van der Waals interaction potential depends exclusively on the distance between sites, we introduce correlations through a translationally invariant Jastrow factor^50^. This choice of parametrization has the advantage of requiring a reduced number of parameters, scaling only linearly with the system size. We find that this ansatz is a good compromise between expressivity and the induced computational burden of time evolution. Indeed, our ansatz does not impose any restriction on the length of the correlations that can be captured by the model and is therefore compatible with the topological character of a QSL. Its functional form is given by

7 $${\psi }_{{\mathbf{\uptheta }}}^{\;{\rm{JMF}}}({\mathbf{\upsigma }})=\exp \left(\sum _{i < j}{\sigma }_{i}{V}_{{d}_{ij}}{\sigma }_{j}\right)\prod _{i}{\varphi }_{i}({\sigma }_{i}),$$

where *d*_*ij*_ denotes the Euclidean distance between any two sites *i* and *j*, and *σ*_*i*_ ∈ {↑, ↓} the spin of the atom at site *i*. Here, the mean-field values *φ*_*i*_(↑), *φ*_*i*_(↓) as well as the Jastrow potential *V*_*d*_ play the role of variational parameters.

This representation is able to faithfully represent an RVB state. Indeed,

8 $$\begin{array}{rcl}\left\vert {\rm{RVB}}\right\rangle &\propto &\prod\limits_{v\in {\mathcal{V}}}\left(\prod\limits_{i\in E(v)}{\hat{n}}_{i}\prod\limits_{\begin{array}{c}j\in E(v)\\ j\ne i\end{array}}(1-{\hat{n}}_{j})\right){\left\vert +\right\rangle }^{\otimes N}\\&\propto &\mathop{\lim }\limits_{W\to -\infty }\exp \left[W\sum\limits_{i < j}{\hat{n}}_{i}{\chi }_{ij}{\hat{n}}_{j}-W\sum\limits_{i}{\hat{n}}_{i}\right]{\left\vert +\right\rangle }^{\otimes N}\\ &\propto &\mathop{\lim }\limits_{W\to -\infty }\exp \left[\frac{W}{4}\sum\limits_{i < j}{\hat{\sigma }}_{i}^{z}{\chi }_{ij}{\hat{\sigma }}_{j}^{z}\right]\times \exp \left[-\frac{W}{2}\sum\limits_{i}({z}_{i}-1){\hat{\sigma }}_{i}^{z}\right]{\left\vert +\right\rangle }^{\otimes N},\end{array}$$

where |+〉 = (|↑〉 + |↓〉)/√2, ${\mathcal{V}}$ denotes the set of vertices of the Kagome lattice, *E*(*v*) refers to the set of all sites *i* at edges connected to the vertex *v*, *χ*_*ij*_ ∈ {0, 1} indicates if the two sites *i* and *j* are connected to the same vertex and *z*_*i*_ = ∑_*j*_*χ*_*ij*_ is the number of sites sharing a vertex with *i*. While the limiting process can seem ill defined whenever ${\sigma }_{i}^{z}{\chi }_{ij}{\sigma }_{j}^{z}=-1$, we highlight the fact that the normalization of the wave function, neglected here, lifts such concerns. The final expression has a two-body Jastrow form fully compatible with our ansatz (equation (7)).

### Monte Carlo estimates

The Born distribution *p*(**σ**) = ∣*ψ*_**θ**_(**σ**)∣^2^/〈*ψ*_**θ**_∣*ψ*_**θ**_〉 can be sampled using the Markov chain Monte Carlo method. The expectation value of any local or sparse operator $\hat{O}$ is efficiently approximated using Monte Carlo integration $\langle \hat{O}\rangle ={{\mathbb{E}}}_{{\mathbf{\upsigma }} \sim p}\left[{O}_{{\rm{loc}}}({\mathbf{\upsigma }})\right]$ with a local estimator given by${O}_{{\rm{loc}}}({\mathbf{\upsigma }})={\sum }_{{{\mathbf{\upsigma }}^{{\prime} }}} \langle {\mathbf{\upsigma }}\vert \hat{O}\vert {{\mathbf{\upsigma }}}^{{\prime} }\rangle \frac{{\psi }_{{\mathbf{\uptheta }}}({{\mathbf{\upsigma }}}^{{\prime} })}{{\psi }_{{\mathbf{\uptheta }}}({\mathbf{\upsigma }})}$, where the sum runs over the few configurations **σ**′ for which the matrix elements $\langle {\mathbf{\upsigma }}\vert \hat{O}\vert {{\mathbf{\upsigma }}}^{{\prime} }\rangle$ are non-zero.

The Rényi-2 entanglement entropy of a given wave function *ψ* is estimated by sampling two replicas of the system^45,51,52^. For each replica, we partition the sites into two regions, $X={\{\left\vert \uparrow \right\rangle ,\left\vert \downarrow \right\rangle \}}^{\otimes {N}_{X}}$ and its complement $Y={\{\left\vert \uparrow \right\rangle ,\left\vert \downarrow \right\rangle \}}^{\otimes (N-{N}_{X})}$, and evaluate

9 $${S}_{X}^{(2)}=-\ln \left[{{\mathbb{E}}}_{\begin{array}{c}{{\mathbf{\upsigma }}} \sim | \psi {| }^{2}\\ {{\mathbf{\upsigma }}}^{{\prime} } \sim | \psi {| }^{2}\end{array}}\left[\frac{\psi ({{\mathbf{\upsigma }}}_{X}^{{\prime} },{{\mathbf{\upsigma }}}_{Y})\psi ({{\mathbf{\upsigma }}}_{X},{{\mathbf{\upsigma }}}_{Y}^{{\prime} })}{\psi ({{\mathbf{\upsigma }}}_{X},{{\mathbf{\upsigma }}}_{Y})\psi ({{\mathbf{\upsigma }}}_{X}^{{\prime} },{{\mathbf{\upsigma }}}_{Y}^{{\prime} })}\right]\right],$$

where ${{\mathbf{\upsigma }}}_{X},{{\mathbf{\upsigma }}}_{X}^{{\prime} }\in X$ and ${{\mathbf{\upsigma }}}_{Y},{{\mathbf{\upsigma }}}_{Y}^{{\prime} }\in Y$ are configurations in either region of the lattice and ${{\mathbf{\upsigma }}}^{({\prime} )}=({{\mathbf{\upsigma }}}_{X}^{({\prime} )},{{\mathbf{\upsigma }}}_{Y}^{({\prime} )})\in {\mathscr{H}}$ form the basis states of the full Hilbert space.

To directly extract the TEE numerically, we adopt the Kitaev–Preskill prescription^32^, where three regions A, B and C converge at a triple intersection point forming a disc, as depicted in Fig. 1. The TEE is then expressed as the following linear combination:

10 $$-\gamma ={S}_{\mathrm{A}}+{S}_{\mathrm{B}}+{S}_{\mathrm{C}}-{S}_{\mathrm{AB}}-{S}_{\mathrm{BC}}-{S}_{\mathrm{CA}}+{S}_{\mathrm{ABC}},$$

where *S*_*XY*…_ denotes the entanglement entropy of the composite region *X* ∪ *Y* ∪ …. This approach offers several advantages, including the absence of linear extrapolation to eliminate area-law terms. Additionally, it entails solely contractible entanglement boundaries (provided that no partition intersects a physical boundary), eliminating the reliance on the state decomposition (see ref. ^53^, where the TEE is contingent upon the minimal-entropy state decomposition of the wave function).

### t-VMC

The time-dependent variational principle (TDVP)^28,54^ evolves the variational state by varying its parameters **θ**. The equation of motion is obtained through the minimization of the Fubini–Study distance between a state with new parameters $\left\vert {\psi }_{{\mathbf{\uptheta }}(t)+{\dot{\mathbf{\uptheta }}}\,\updelta t}\right\rangle$ and the evolved state *U*(δ*t*)∣*ψ*_**θ**(*t*)_〉. The resulting equation

11 $$\sum _{{k}^{{\prime} }}{S}_{k{k}^{{\prime} }}{\dot{\theta }}_{{k}^{{\prime} }}(t)=-i{C}_{k}$$

depends on two quantities that can be sampled using Monte Carlo, resulting in the t-VMC prescription^28,55^. First, the quantum geometric tensor describes the covariance of the gradients of the wave function:

12 $$\begin{array}{rcl}{S}_{ij}&=&\frac{\left\langle {\partial }_{i}\psi \vert {\partial }_{j}\psi \right\rangle }{\langle \psi \vert \psi \rangle }-\frac{\left\langle {\partial }_{i}\psi \vert \psi \right\rangle \left\langle \psi \vert {\partial }_{j}\psi \right\rangle }{{\langle \psi \vert \psi \rangle }^{2}}\\ &=&{\mathbb{E}}\left[{D}_{i}^{* }({D}_{j}-{\mathbb{E}}[{D}_{j}])\right],\end{array}$$

where *D*_*k*_(**σ**) = ∂_*k*_ log *ψ*(**σ**) can be obtained by automatic differentiation^56^. Second, the vector of forces represents the derivatives of the energy in the same parameter space:

13 $$\begin{array}{rcl}{C}_{i}&=&\frac{\left\langle {\partial }_{i}\psi \right\vert \hat{{\mathcal{H}}}\left\vert \psi \right\rangle }{\langle \psi \vert \psi \rangle }-\frac{\left\langle {\partial }_{i}\psi \vert \psi \right\rangle \left\langle \psi \right\vert \hat{{\mathcal{H}}}\left\vert \psi \right\rangle }{{\langle \psi \vert \psi \rangle }^{2}}\\ &=&{\mathbb{E}}[{D}_{i}^{* }({E}_{{\rm{loc}}}-{\mathbb{E}}[{E}_{{\rm{loc}}}])],\end{array}$$

where we introduced the local energy *E*_loc_.

### Mean-field evolution

As recently shown^57^, the Monte Carlo estimators defined in equations (12) and (13) are strongly biased whenever the amplitude of the variational wave function vanishes for basis states yielding finite contributions to the gradients. This is a generic feature for distributions with many zeros, such as the initial state in our case. To circumvent this issue, we evolve the initial state by analytically solving the TDVP equations, that is, dispensing with any sampling, on a simplified ansatz with all Jastrow parameters set exactly to zero:

14 $$\left\vert {\psi }_{{\mathbf{\uptheta }}}\right\rangle =\mathop{\bigotimes }\limits_{i=1}^{N}\left({\alpha }_{i}{\left\vert \uparrow \right\rangle }_{i}+{\beta }_{i}{\left\vert \downarrow \right\rangle }_{i}\right),$$

where we use the correspondence |*g*〉 = |↑〉 and |*r*〉 = |↓〉 and consider normalized parameters (∣*α*∣^2^ + ∣*β*∣^2^ = 1). The product-state nature of the ansatz implies that *S* is block diagonal, only coupling the parameters *β*_*k*_, *α*_*k*_ acting on the same site. Thus, we only need to solve a two-dimensional linear equation. Upon neglecting the indices *k* and time *t* for the forces to simplify notations, and defining the force amplitudes *C*_*β*_ = *B* and *C*_*α*_ = *A*, we obtain the system

15 $$\left(\begin{array}{cc}| \alpha {| }^{2}&-\beta {\alpha }^{* }\\ -{\beta }^{* }\alpha &| \beta {| }^{2}\end{array}\right)\left(\begin{array}{c}\dot{\beta }\\ \dot{\alpha }\end{array}\right)=-i\left(\begin{array}{c}B\\ A\end{array}\right).$$

To decrease the number of degrees of freedom, we fix the global phase by setting *α*^I^ = 0, where superscripts R and I indicate the real and imaginary parts, respectively, and use the normalization constraint to express *α* and $\dot{\alpha }$ in terms of the other two parameters. The system (equation (15)) can then be solved by diagonalizing *S*, replacing ${\dot{\alpha }}^{{\rm{R}}}$ and decomposing into real and imaginary parts:

16 $$\begin{array}{rcl}{\dot{\beta }}^{{\rm{R}}}&=&+({({\alpha }^{{\rm{R}}})}^{2}+{(\;{\beta }^{{\rm{I}}})}^{2}){B}^{{\rm{I}}}+{\beta }^{{\rm{I}}}{\beta }^{{\rm{R}}}{B}^{{\rm{R}}}-{\alpha }^{{\rm{R}}}{\beta }^{{\rm{R}}}{A}^{{\rm{I}}}-\frac{1}{{\alpha }^{{\rm{R}}}}{\beta }^{{\rm{I}}}{A}^{{\rm{R}}},\\ {\dot{\beta }}^{{\rm{I}}}&=&-({({\alpha }^{{\rm{R}}})}^{2}+{(\;{\beta }^{{\rm{R}}})}^{2}){B}^{{\rm{R}}}-{\beta }^{{\rm{I}}}{\beta }^{{\rm{R}}}{B}^{{\rm{I}}}-{\alpha }^{{\rm{R}}}{\beta }^{{\rm{I}}}{A}^{{\rm{I}}}+\frac{1}{{\alpha }^{{\rm{R}}}}{\beta }^{{\rm{R}}}{A}^{{\rm{R}}}.\end{array}$$

Notice that in these equations the division could be ill defined if *α*^R^ = 0. Fortunately, this will never be the case in our situation, since (*α*^R^)^2^ is the probability of having the site *k* in |*g*〉, which is always close to 1 for *Δ* < 0 (in practice, this is true throughout the whole evolution).

We find that the particles are effectively subject to a local potential $v=-\varDelta +\varOmega {\sum }_{l\ne k}{(\frac{{R}_{\mathrm{b}}}{{r}_{kl}})}^{6}| {\beta }_{l}{| }^{2}$. Then, the forces are given by

17 $$\begin{array}{rcl}B&=&-\frac{\varOmega }{2}\left(\alpha -2\beta {\beta }^{{\rm{R}}}\alpha \right)+v\beta | \alpha {| }^{2},\\ A&=&-\frac{\varOmega }{2}\left(\beta -2\alpha {\beta }^{{\rm{R}}}\alpha \right)-v\alpha | \beta {| }^{2},\end{array}$$

where we evaluate the frequencies at the same time step. Putting everything together, we obtain

18 $$\begin{array}{rcl}{\dot{\beta }}^{{\rm{R}}}&=&+v{\beta }^{{\rm{I}}}-\frac{\varOmega }{2}\left(-\frac{{\beta }^{{\rm{I}}}{\beta }^{{\rm{R}}}}{\alpha }\right),\\ {\dot{\beta }}^{{\rm{I}}}&=&-v{\beta }^{{\rm{R}}}-\frac{\varOmega }{2}\left(\frac{{(\;{\beta }^{{\rm{R}}})}^{2}}{\alpha }-\alpha \right).\end{array}$$

Finally, exploiting again the normalization and gathering *β* = *β*^R^ + *iβ*^I^, we obtain the final solution for the complex parameters:

19 $$\begin{array}{rcl}{\dot{\beta }}_{k}(t)&=&-i{v}_{k}(t){\beta }_{k}-i\frac{\varOmega (t)}{2}\left({\beta }_{k}\frac{{\beta }_{k}^{{\rm{R}}}}{{\alpha }_{k}}-{\alpha }_{k}\right),\\ {\dot{\alpha }}_{k}(t)&=&-\frac{\varOmega (t)}{2}{\beta }_{k}^{\mathrm{I}}.\end{array}$$

This analytical evolution is conducted for a short starting time t^*^ < *T*, up to a point where the distribution has spread over more basis states and we can use the Markov chain Monte Carlo estimate of the TDVP equation. Typically, we set this value to ${t}^{* }=\frac{2}{25}T$, corresponding to 40% of the time used for the sweeping of the Rabi frequency (see Supplementary Section 1 for details of the protocol).

### Accuracy of simulations

To better understand the validity of our variational approach, we investigate the main candidates for numerical error in the t-VMC prescription. For this purpose, we consider a small system on which exact calculations can be performed. Thus, we use a lattice of *N* = 24 sites, composed of one and a half hexagons. To mitigate finite-size effects, we take both boundaries to be periodic.

**Extended Data Fig. 6:**
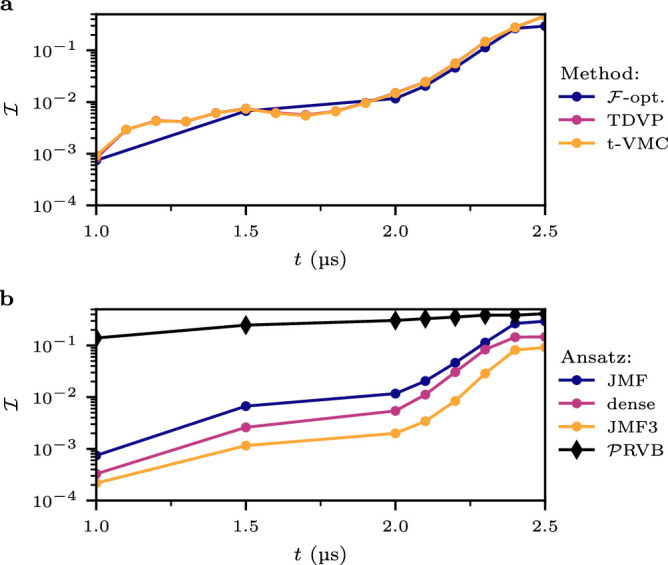
Infidelity of the simulated state compared to exact evolution. Infidelity ${\mathcal{I}}(t)=1-| \left\langle {\psi }_{{\rm{exact}}}(t) \vert {\psi }_{{\boldsymbol{\theta }}}(t)\right\rangle {| }^{2}$ of the variational state at all times during the time evolution. All data is obtained on a small lattice (*N* = 24) defined in Methods in order to compare with the exactly evolved state *ψ*_exact_(*t*) and to compute the infidelity between the states exactly as an inner-product. **a** Comparison of the ${\psi }_{{\boldsymbol{\theta }}}^{\;{\rm{JMF}}}$ Ansatz for various integration schemes, namely fidelity optimization w.r.t. the exact solution (dark blue), exact evolution of the TDVP equation of motion without any sampling (purple) and the usual time-dependent variational Monte Carlo (yellow). **b** Comparison of multiple ansätze trained through fidelity optimization. The ansätze are the JMF equation (7), the dense Jastrow equation (20), the three-body Jastrow equation (21) and the projected RVB equation (22) presented in Methods. Source data

First, we want to ensure that t-VMC converges to the correct state. For this purpose, we compare the fidelity with the exact state at all times for three different numerical schemes: (1) fidelity optimization of our ansatz with respect to the reference state carried out at every time (no time evolution), (2) TDVP (no Monte Carlo sampling) and (3) t-VMC as in the main text. We first observe in Extended Data Fig. 6a that the three schemes considered qualitatively yield the same results, with an excellent fidelity at small times, which then increases around *t* = 2 μs. The discrepancy between TDVP and t-VMC is practically indiscernible, which confirms that our Markov chain Monte Carlo sampling scheme is not a notable source of numerical error. Moreover, the comparison with fidelity optimization allows to rule out the rest of the t-VMC scheme as a potential source of error, in particular the discretization of the dynamical equations and the inversion of the quantum geometric tensor at each step. Thus, calculating and solving the TDVP equation of motion does not increase the infidelity during the simulation.

Therefore, the only source left to verify is the expressivity of the ansatz itself. With this aim, we compare ansätze with varying design and assess whether this has an important impact on the fidelity. We restrict ourselves to ansätze within the Jastrow class since they allow for a great numerical stability.

The first additional architecture we consider is the usual dense (all-to-all) Jastrow obtained by replacing the invariant parameters of our ansatz by a dense matrix as ${V}_{{d}_{ij}}\to {W}_{ij}$, leading to

20 $${\psi }_{{\mathbf{\uptheta }}}^{{\rm{dense}}}({\mathbf{\upsigma }})=\exp \left(\sum _{i < j}{\sigma }_{i}{W}_{ij}{\sigma }_{j}\right)\prod _{i}{\varphi }_{i}({\sigma }_{i}).$$

We also consider a more expressive ansatz by adding a three-body Jastrow interaction term to our existing ansatz:

21 $${\psi }_{{\mathbf{\uptheta }}}^{\;{\rm{JMF3}}}({\mathbf{\upsigma }})=\exp \left(\sum _{i < j < k}{W}_{{d}_{ij},{d}_{\!jk}}{\sigma }_{i}{\sigma }_{j}{\sigma }_{k}\right)\times {\psi }_{{\mathbf{\uptheta }}}^{\;{\rm{JMF}}}({\mathbf{\upsigma }}).$$

Notice that this form is again a translationally invariant Jastrow, supplemented with a mean field. It was shown^55^ that *M*-body Jastrow ansätze are able to represent states up to a residual involving correlations of order >*M*. Hence, this ansatz should perform better than the two-body ansatz. However, it introduces a substantial numerical overhead, as the number of parameters scales quadratically with the system size *N* instead of linearly.

Furthermore, another form of variational ansatz, which can easily represent the RVB state, has been recently proposed in ref. ^36^. The corresponding wave function is given by

22 $$\left\vert {\psi }_{{\mathbf{\uptheta }}}^{{\mathcal{P}} {\rm{RVB}}}\right\rangle =\bigotimes _{i}\left(1+{z}_{2}{\hat{\sigma }}_{i}^{+}\right)\left(1+{z}_{1}{\hat{\sigma }}_{i}^{-}\right)\left\vert {\rm{RVB}}\right\rangle ,$$

where the RVB state is an equal-weight superposition of all defect-free dimerizations of the lattice. The operators are respectively ${\hat{\sigma }}_{i}^{-}=| {g}_{i}\left.\right\rangle \left\langle \right.{r}_{i}|$ and ${\hat{\sigma }}_{i}^{+}=| {r}_{i}\left.\right\rangle \left\langle \right.{g}_{i}|$ tuned by the complex parameters *z*_1_ and *z*_2_. By setting *z*_1_ = 0 = *z*_2_, we obtain exactly the sought-after RVB state, which can faithfully be implemented using tensor networks on single vertices and projecting out non-valid dimerizations^36^. Whenever *z*_1_, *z*_2_ ≠ 0, this ansatz requires the application of a dense matrix to the RVB state. However, Monte Carlo calculations are efficient only for sparse or local operators and thus this ansatz is not scalable to large systems.

To analyse representational power isolated from any other source of numerical error, these ansätze are all optimized by minimizing the infidelity to the exact solution ${\mathcal{I}}(t)=1-| \left\langle {\psi }_{{\rm{exact}}}(t) \vert {\psi }_{{\boldsymbol{\theta }}}(t)\right\rangle {| }^{2}$ at all times. The optimizations were carried out on the small lattice of *N* = 24 dispensing from any Monte Carlo sampling.

We deduce from the results in Extended Data Fig. 6b that the qualitative behaviour between a dense Jastrow ansatz and an invariant one is the same. While the dense Jastrow has a generally higher fidelity, the JMF ansatz allows for a great reduction in the number of parameters without substantially impacting the state. This is due to the fact that we include an additional inhomogeneous mean-field part, which breaks any spatial translational invariance. As expected, the addition of a three-body term increases the expressivity of the ansatz and reduces even further the infidelity, even though the ansatz uses invariant parameters (as opposed to the dense Jastrow). For approximately the same number of parameters, the partially invariant three-body Jastrow obtains better results than a dense two-body ansatz. This reinforces the idea that, upon increasing the order of the Jastrow, the infidelity of the prepared state can be arbitrarily reduced.

In contrast, even though the ${\mathcal{P}}{\rm{RVB}}$ performs comparably to the Jastrow ansätze at large times, its representativity is notably worse at intermediate times. Thus, considering t-VMC, the use of this ansatz would accumulate errors throughout the evolution. Therefore, this ansatz, though useful in other contexts^36^, is unsuitable for the simulation of dynamical preparation protocols from trivial initial states.

All considered ansätze can represent exactly the RVB state. For the ${\mathcal{P}}{\rm{RVB}}$ ansatz, this is achieved for *z*_1_ = 0 = *z*_2_, while for the two-body Jastrow this is obtained in the limit *W* → −*∞* (see equation (8)). Still, none reach vanishing infidelities at the final times of the simulation of the state preparation. Therefore, we conclude that the final states are different from the RVB state. Thus, when designing an expressive ansatz for this task, it is not only key for it to be able to represent the RVB state, but also to be able to capture the departure from it induced by the long-range tails in the van der Waals potential which ultimately produces a correlated phase resembling a topologically ordered phase at times *t* > 2.0 μs. In general, we conclude that our choice of two-body translation-invariant Jastrow with inhomogeneous mean field yields a sufficiently high representational power to represent all states encountered during the time evolution.

### VMC study of the toric-code model

Unlike the rest of this work, the order of the perturbed toric-code model can be probed via standard VMC simulations. The parameters of the variational wave function *ψ*_**θ**_ are optimized to minimize the energy. This can be achieved through stochastic gradient descent,

23 $${{\mathbf{\uptheta }}}^{i+1}={{\mathbf{\uptheta }}}^{i}-\eta {\boldsymbol{\nabla }}\langle \hat{{\mathcal{H}}}\rangle ,$$

where the energy gradient ${\partial }_{k}\langle \hat{{\mathcal{H}}}\rangle ={\mathbb{E}}[\left({E}_{{\rm{loc}}}({\bf{x}})-{\mathbb{E}}\left[{E}_{{\rm{loc}}}({\bf{x}})\right]\right){D}_{k}^{\star }({\bf{x}})]$ can be efficiently estimated via Monte Carlo integration. Here *i* indicates the current optimization step, and the learning rate *η* = 10^−3^ is a free hyperparameter, which sets the magnitude of each gradient-descent update. For a better stability and exponential guarantees of convergence, stochastic reconfiguration is used^58^, where the parameter gradient is preconditioned as δ**θ** = *S*^−1^**C** to match the imaginary time-evolution update, similarly to equation (11).

Motivated by the capability of the Jastrow wave function to exactly represent the RVB state (see above), we pursue this scheme using our Jastrow ansatz. Even though this analysis is solely conducted on a toric lattice, which should ensure translational invariance, we choose to use the more general, site-dependent form of the Jastrow correlator, as presented in equation (20), since a higher number of parameters can be used in VMC calculations without facing any numerical issues.

Importantly, in the presence of external fields, the restriction of the Hilbert space introduced above is no longer justified and has to be dropped. Therefore, a new Markov chain Monte Carlo sampling scheme, which can be efficient within the restricted Hilbert space but also allows us to enter and leave this space, is required. Given that the $\hat{Q}$ operator maps any correct dimerization to another valid one when acting on a closed string, it can be used as a Metropolis–Hastings transition rule to efficiently sample the RVB state. In addition to this, local rules are necessary to ensure that the sampling scheme be ergodic. At every Metropolis–Hastings step, we choose to apply the $\hat{Q}$ operator on a randomly drawn closed hexagon with a probability of 75%, applying it on a random site with 12.5% and to simply flip a single spin with 12.5%, which allows for efficient yet ergodic sampling of the full Hilbert space.

Finally, the initialization of the process is central to achieve a fast convergence. At zero field, the ground state of the system is simply the RVB state, which can be represented with our variational wave function in some limiting regime (see above). Thus, at low field, the RVB is guaranteed to be close to the aimed state. However, initializing parameters to large values ∣*W*∣ ≫ 1 results in vanishing gradients and longer optimization procedures. Therefore, our state is initialized with a parameter structure close to that producing the RVB state, yet with much lower parameter amplitudes. More specifically, parameters that yield an RVB when taking their magnitude to infinity are here initialized at a large, yet finite value, while the rest of the parameters are randomly drawn close to zero.

## Online content

Any methods, additional references, Nature Portfolio reporting summaries, source data, extended data, supplementary information, acknowledgements, peer review information; details of author contributions and competing interests; and statements of data and code availability are available at 10.1038/s41567-025-02944-3.

## Supplementary information

Supplementary Information Supplementary Discussion.

## Source data

Source Data Fig. 2 Statistical source data.

Source Data Fig. 3 Statistical source data.

Source Data Fig. 4 Statistical source data.

Source Data Extended Data Fig. 1 Statistical source data.

Source Data Extended Data Fig. 2 Statistical source data.

Source Data Extended Data Fig. 3 Statistical source data.

Source Data Extended Data Fig. 4 Statistical source data.

Source Data Extended Data Fig. 5 Statistical source data.

Source Data Extended Data Fig. 6 Statistical source data.

## Data Availability

The numerical data supporting this study are available via Zenodo at 10.5281/zenodo.13318731 (ref. ^59^). Source data are provided with this paper.

## Code availability

The simulations in this work were carried out using NetKet^60,61^, which is based on JAX^56^ and MPI4Jax^62^. The code used to generate all data in this study is available via Zenodo at 10.5281/zenodo.13318731(ref. ^59^).
