# Supplementary material for: Predicting topological entanglement entropy in a Rydberg analogue simulator
Source: Nat Phys. 2025 Jul 28;21(8):1332–7. doi: 10.1038/s41567-025-02944-3 (PMC12343300; doi:10.1038/s41567-025-02944-3)
Supplement: Supplementary file 1 — Supplementary Discussion. [file 41567_2025_2944_MOESM1_ESM.pdf]

nature physics

Supplementary information https://doi.org/10.1038/s41567-025-02944-3

Predicting topological entanglement entropy in a Rydberg analogue simulator

In the format provided by the authors and unedited

Supplementary Information

Contents

S1 Influence of the protocol on the results **1**

S2 Logical operations **1**
S2.1 Definition . . . 2
S2.2 Pseudo-identity . . . 2
S2.3 Pauli algebra . . . 2

S3 Stochastic unraveling of disordered open quantum systems **3**
S3.1 Algorithm . . . 3
S3.2 Sources of error . . . 3
S3.3 Effect of experimental noise on topological operators . . . 4

S4 Lattice topology and finite size effects **5**

S1 Influence of the protocol on the results

As discussed in the main text, to approach the QSL state, a delicate balance of adiabaticity must be used in the protocol of preparation. Here, we will study the influence of the choice of protocol on the type of states reached during the state preparation. In this respect, we consider a family of similar protocols as depicted on Extended Data Fig. 1, where the detuning increases as a polynomial function of the time after the Rabi frequency $\Omega(t)$ has attained its maximal value Ω_0. While an unlimited amount of different protocols could be considered, through the modification of the initial and final values of both frequencies, the total sweeping time or the functional shape of each sweep, we are here only interested in ruling-out the protocol as the source for the lack of topological order by considering similar schedules.

At small times, we observe essentially no difference between the results of the different protocols presented in Extended Data Fig. 1. However, from $\Delta/\Omega_0 = 3$, the behaviors of both $\langle \hat{P} \rangle$ and $\langle \hat{Q} \rangle$ start shifting apart. In particular, we observe that the quadratic protocol does not reach a value as high as the linear and cubic protocols do. However, since one should get exactly $\langle \hat{Q} \rangle = 1$ for a QSL state, no protocol reaches the aimed state.

The analysis of the FM-order parameters highlights the similarity between all the results. Indeed, for all protocols considered, $\langle \hat{P}_{\mathrm{FM}} \rangle$ vanishes from $\Delta/\Omega_0 = 3$. Furthermore, even though the exact values of $\langle \hat{Q}_{\mathrm{FM}} \rangle$ differ slightly, their behaviors are qualitatively similar among all protocols. In particular, $\langle \hat{Q}_{\mathrm{FM}} \rangle$ vanishes for all considered cases, yet the vanishing point is shifted for the linear protocol.

While this analysis is not based on global topological metrics such as the topological entanglement entropy, the information brought by the topological operators already gives a valuable indication that small changes in the protocols have no significant impact on the reachable states. We leave a thorough analysis of the optimization of the protocol to maximize the value of γ to future work.

S2 Logical operations

As we observe throughout this work, the prepared state seems to exhibit features that locally resemble those found in the surface code. A further salient characteristic of such a code is the condensation of anyons at the physical boundaries of the lattice. For this reason, the topology of the simulated system has a crucial impact on this phenomenon. We here investigate this by adding a hole in the bulk of a large planar lattice ($N = 285$ atoms), thereby creating a second physical boundary [1, 2]. This induces condensation of m anyons on both the external boundary and the hole, separating the Hilbert space of dimer coverings into two topological sectors, which can be labeled as logical quantum

bits $|0\rangle_L$ and $|1\rangle_L$, as introduced by ref. [1]. While this approach poses severe challenges for DMRG-based methods due to the shape of the lattice, the flexibility of our method allows for a complete study of this system.

S2.1 Definition

In this framework, we define logical operators $\hat{X}_L$ and $\hat{Z}_L$ acting on the logical states $|0\rangle_L$ and $|1\rangle_L$,

$$\begin{aligned} \hat{X}_L |0\rangle_L &= |1\rangle_L\,, \quad \hat{Z}_L |0\rangle_L = |0\rangle_L\,, \\ \hat{X}_L |1\rangle_L &= |0\rangle_L\,, \quad \hat{Z}_L |1\rangle_L = -|1\rangle_L\,, \end{aligned} \tag{1}$$

defined along paths illustrated in Extended Data Fig. 2**a**. The off-diagonal operator $\hat{X}_L$ maps one logical state to the other and is applied to a path that circumvents the hole. The diagonal operator $\hat{Z}_L$ allows for the identification of the spin projection of the logical qubit and is applied to a path that connects the two separated boundaries. The definition of both logical operators roots in the anyonic properties of the vacuum ground state of the toric code. In the anyonic picture, $\hat{Z}_L$ acts by connecting together m-anyons on both boundaries. Also, $\hat{X}_L$ acts by creating an e-anyon, winding it around the m-anyons condensed on the hole, and finally destroying it. Therefore, both operators can be defined in multiple ways, as long as the topology of the path they are applied on remains unchanged.

Locally, these logical operators act the same way as the previously defined topological operators $\hat{Q}$ and $\hat{P}$. As a consequence, the sign of the expectation value of any closed diagonal operator will solely be dictated by the parity of the number of vertices enclosed by its path [2], namely $(-1)^{\#\text{vertices}}$. On the other hand, we showed in previous analyses that both $\langle \hat{P} \rangle$ and $\langle \hat{Q} \rangle$ reached reduced values compared to a perfect QSL in the simulation of the Rydberg experiment. As the magnitude of topological operators is affected by the presence of defects, we expect the same behavior to occur for the logical operators. In that case, the previously stated properties of the logical operators $\hat{X}_L$ and $\hat{Z}_L$ are only approximate and the exact impact on the results is to be determined.

Equipped with these operators, it is possible to identify the state prepared on the logical qubit. We find $\langle \hat{X}_L \rangle > 0$ and $\langle \hat{Z}_L \rangle \sim 0$ in the approximate dimer phase, identifying our state with an approximate logical state $|\psi\rangle \sim |+\rangle_L = (|0\rangle_L + |1\rangle_L)/\sqrt{2}$ with additional defects. Despite the topology being different from the lattice studied in the previous section, the prepared state is also an approximate superposition of dimerizations from both topological sectors, thus approaching the RVB state in the same fashion as for the planar lattice without a hole defined in Fig. 1. Moreover, the condensation of m-anyons at both boundaries is confirmed by the expectation values of both logical operators.

S2.2 Pseudo-identity

We further use the Pauli identity $\hat{\mathbb{1}} = \hat{Z}_L \times \hat{Z}_L$ that holds for perfect topological qubits and detect deviations from this identity to characterize the imperfect QSL. In a state with perfect topological invariance of the logical operators, the identity holds more generally (up to a sign) when we consider the product of two different logical operators $\hat{Z}_L^{(1)}\hat{Z}_L^{(j)}$. However, the parity is non-trivial and depends entirely on the number of vertices enclosed between the two strings through $(-1)^{\#\text{vertices}}$. We will therefore evaluate the identity at increasing separation distance between the two strings on which the $\hat{Z}_L^{(j)}$ operators are defined.

We numerically verified the expected parity sign structure. We show the absolute values of the desired identity for multiple pairs of paths that make up loops that contain an increasing number of sites in Extended Data Fig. 2**b**. The qualitative behavior is similar for all loops and confirms a finite expectation value at late times, when a QSL-like phase was diagnosed in the dynamic state preparation. However, a significant difference appears in the magnitude of the different observables. We observe that, as the two operators $\hat{Z}_L^{(1)}$ and $\hat{Z}_L^{(j)}$ are further away from each other, $\left|\langle \hat{Z}_L^{(1)}\hat{Z}_L^{(j)} \rangle\right|$ decreases. This observation confirms that, even though the prepared state presents local features of a QSL, it contains non-trivial differences from an RVB state that clearly appear in larger-scale observables.

S2.3 Pauli algebra

As we only have access to the dynamically prepared state $|\psi\rangle \sim |+\rangle_L$, it is not possible to fully characterize the logical algebra we defined. However, as a first step to verify the structure of Lie algebra, we evaluate the identity $\{\hat{X}_L, \hat{Z}_L\} = 0 \iff \hat{X}_L = -\hat{Z}_L \times \hat{X}_L \times \hat{Z}_L$. In experiments, the verification of this identity poses serious challenges, since the observables to be observed act on a large number of sites and consist of both diagonal and non-diagonal operators. As previously shown, the interplay between experimental noise and the basis rotation involved in the measurement of the latter yields sizable spurious effects.

As in the study of the identity in the previous subsection, the use of a closed loop composed of two separate $\hat{Z}_L$ is required for the operator to be non-trivial. Here also, a parity factor appears that depends on the number of

vertices enclosed between the $\hat{Z}_L$ operators. We verified that the sign of $\langle \hat{Z}_L^{(1)} \hat{X}_L \hat{Z}_L^{(j)} \rangle$ corresponds to $-(-1)^{\#\text{vertices}}$, and thus is exactly opposite to the sign of the pseudo identity $\langle \hat{Z}_L^{(1)} \hat{Z}_L^{(j)} \rangle$. Given that the sign of $\langle \hat{X}_L \rangle$ is always positive, we show the absolute value of the rotated $\hat{X}_L$ in Extended Data Fig. 2**c**. Before the onset of the topologically ordered phase around $t = 2.2\,\mu\text{s}$, we observe that $|\langle \hat{X}_L \rangle|$ and its rotated counterpart $|\langle \hat{Z}_L^{(1)} \hat{X}_L \hat{Z}_L^{(j)} \rangle|$ qualitatively differ. In contrast, at later times when the system exhibits topological order, the qualitative behavior of all curves agrees for all realizations of the logical operator $\hat{Z}_L$ defined on any of the tested topologically equivalent paths. We find that the further apart the operators $\hat{Z}_L^1$ and $\hat{Z}_L^j$ are, the poorer the agreement. This allows us to confirm that the operators approximately respect the Pauli algebra at small distances and thus approximately behave as logical operators. Deviations in the expectation value of these observables are again imputable to defects.

The analysis conducted in this section is a striking illustration of the strengths of our method, namely (i) its flexibility in terms of geometry and topology, and (ii) its ability to directly measure string operators, whether diagonal or not. A thorough study of the anyonic properties of topologically ordered states by evaluating products of non-commuting string operators should entail a prohibitive overhead for experiments, which would require subsequent rotations of the local basis, which results in a significant source of noise and errors (see next section).

S3 Stochastic unraveling of disordered open quantum systems

In the main text, we are considering the dynamical preparation of a QSL state using a Rydberg Hamiltonian from first principles. However, a state prepared on a real-world quantum device might differ from numerical simulations non-trivially due to the spurious effects of noise throughout state preparation. While t-VMC prescription solves the time-dependent Schrödinger equation of a closed quantum system, in the event of interactions with the environment, the latter needs to be treated within the formalism of open quantum system [3]. This can be done through a Lindblad master equation. Such an exact description implies the time evolution of the density matrix $\hat{\rho}$ under a Liouvillian map. The complexity of this evolution scales quadratically worse than that of $|\psi\rangle$, making the approach intractable for most use cases, in particular in the present one which involves over 200 Rydberg atoms. To circumvent this, we make use of the quantum trajectories prescription, which expresses the solution of the master equation $\hat{\rho}$ as an average over multiple random realizations (trajectories) $|\psi\rangle$ of a stochastic Schrödinger equation.

S3.1 Algorithm

To efficiently compute trajectories, we use the faster-than-the-clock algorithm [4, 5] described in what follows. The state of each trajectory is evolved under the pseudo-Hermitian Hamiltonian

$$\hat{\mathcal{H}}^{\text{nh}} = \hat{\mathcal{H}} - \frac{i}{2} \sum_j \hat{L}_j^\dagger \hat{L}_j \,, \tag{2}$$

where the sum runs over all noise channels j with associated jump operators $\hat{L}_j$. Due to the non-Hermitian nature of this Hamiltonian, the norm of the wave function will decrease in time. We make use of this norm to decide when a quantum jump occurs. For each trajectory, a random number $\eta \in [0, 1]$ is drawn and once the norm reaches $||\psi(t)||^2 = 1 - \eta$, a quantum jump occurs. The jump is randomly picked from all the existing channels with respective probabilities $p_j(t) = \langle \hat{L}_j^\dagger \hat{L}_j \rangle / \eta$. The state after a jump is given by

$$\left|\psi(t^+)\right\rangle = \frac{1}{\sqrt{\eta p_j(t^-)}} \hat{L}_j \left|\psi(t^-)\right\rangle \,, \tag{3}$$

which results in a normalized wave function. After the state is projected, a new number η is drawn, corresponding to the trigger for the next jump.

In order to evaluate operators on a mixed state, no explicit reconstruction of the density matrix is needed. Instead, one can average the expectation value of the observables of interest over as many stochastic trajectories as needed to reach convergence. In our case where $N = 24$, we use 100 trajectories, each for an independent random realization of the spatial disorder in the parameters of the physical system (see next subsection for details on the modeling of disorder). This procedure optimally reduces the total variance on the trajectory and spatial-disorder average [6].

S3.2 Sources of error

We start by considering inhomogeneities of the Hamiltonian, to represent the spatial disorder in the experiment. This is accounted for as an imprecision in the local fields applied on each atom: $\hat{\mathcal{H}}(t) \to \hat{\mathcal{H}}(t) - \frac{\Omega(t)}{2} \sum_i \omega_i \hat{\sigma}_i^x - \Delta(t) \sum_i \delta_i \hat{n}_i$, where each shift ω_i, δ_i is randomly picked from a normal distribution with chosen variance $\mathcal{N}(0, \sigma^2_{(n,x)})$ [7]. Since we

are considering the errors on the fields to be normally distributed, the impact on observables is reduced as compared to that of systematic errors [8], which we do not consider here. As $\Omega(t)$ is only associated with the kinetic part of the Hamiltonian, inhomogeneities have a small effect on the resulting state. In contrast, modifying the driving field $\Delta(t)$ induces a decoherence effect [9].

Furthermore, to account for the coupling to the environment, we consider the principal error channels, whose jump operators are defined as follows:

$$\begin{aligned} \hat{L}_i^{(z)} &= \sqrt{\kappa^z}\left(|r_i\rangle\langle r_i| - |g_i\rangle\langle g_i|\right) , \\ \hat{L}_i^{(+)} &= \sqrt{\kappa^+}\,|r_i\rangle\langle g_i| , \\ \hat{L}_i^{(-)} &= \sqrt{\kappa^-}\,|g_i\rangle\langle r_i| , \end{aligned} \tag{4}$$

with respective error rates κ^α.

To understand the real impact of each noise channel on the prepared state, we conduct a qualitative analysis presented in Extended Data Fig. 3. Therein, we chose to solely consider diagonal observables in order to avoid the noise effects induced by the basis rotation in the experimental results, which are hard to predict. Firstly, we observe that all noise channels have a similar effect on the quantities shown in Extended Data Fig. 3, namely bringing the results of simulations into closer line with those of experiments, as expected. We observe that out of the three error channels considered, scattering has the strongest impact on the observables. Indeed, as the density of excitations does not exceed $\langle\hat{n}\rangle \approx 1/3$ during the whole time evolution, the application of scattering strongly modifies the mean occupation, and thus, the observables depending on it. By generating excitations randomly in the state, it creates new dimers which do not respect any kind of Rydberg blockade and thus increases the density of double dimers and imperfect dimerizations. On the other hand, as the decay channel destroys excitations, it generates imperfect dimerizations but does not create states violating the Rydberg constraint. Thus, it does not greatly influence the density of defects, but impacts $\langle\hat{P}\rangle$ slightly. Moreover, decoherence has the most difficult effect to predict. As these observables are all diagonal, the influence is reduced compared to $\langle\hat{Q}\rangle$, yet it still contributes to a shift from the exact noiseless results.

Finally, we compare these effects to the experimental measurements obtained in ref. [1]. We find a match between our noisy results and the experimental ones for a value of the scattering rate in agreement with that measured on the experimental setup ($\kappa^+_{\text{exp}} = 1/(150\,\mu\text{s}) \approx 1\,\text{rad}/23.9\,\mu\text{s}$). Notice however that the results of Extended Data Fig. 3 only involve isolated noise channels on a small lattice of $N = 24$ atoms, not the $N = 219$ lattice of the actual experiment.

S3.3 Effect of experimental noise on topological operators

In order to bridge the gap between numerical results in absence of nonidealities and experimental results, one needs to qualitatively assess the effect of noise in a realistic quantum simulator. For this purpose, we consider the realistic error rates of the Rydberg quantum simulator from ref. [1, 7]. In particular, we consider scattering processes at an angular rate of $\kappa^+ = 2\pi/(150\,\mu\text{s})$ and decay at $\kappa^- = 2\pi/(80\,\mu\text{s})$. Furthermore, as previously introduced, we use inhomogeneities in the fields to be of $\sigma_x^2 = 3\%$ for $\Omega(t)$ and $\sigma_n^2 = 2\%$ for $\Delta(t)$. The results obtained for this setting are the ones presented and discussed in Sec. S3.

The simulations in Extended Data Extended Data Fig. 4 show that in both noiseless and noisy simulations, the topological operators present a peak after $t = 2\,\mu\text{s}$, hinting at a transition to a topologically ordered phase. However, as thoroughly discussed in S3.2, the addition of noise to the model has the effect of decreasing all expectation values throughout the evolution. For $\langle\hat{P}\rangle$ and $\langle\hat{Q}\rangle$ in particular, the diminishing of the peaks demonstrates that noise introduces more defects in the dimerizations and that connected dimerizations present less coherence. At short times, in the non-dimerized regime ($\langle\hat{n}\rangle \approx 0$), the behavior of $\langle\hat{P}\rangle$ is significantly impacted by noise. Interestingly, the impact of noise on the FM order parameters is less pronounced than on the topological operators. In fact, while the measurements of topological operators in both open and closed contours are affected by noise, the value of $\langle\hat{Q}\rangle$ is used as a normalization for $\langle\hat{Q}_{\text{FM}}\rangle$. Thus, the reduction of amplitude is greatly compensated for through the ratio of the two expectation values on the open and closed contour, respectively, as intended.

Furthermore, we observe a close agreement between the noisy simulations and the experiment for the diagonal operators $\langle\hat{P}\rangle$ and $\langle\hat{P}_{\text{FM}}\rangle$, while the values for operators $\langle\hat{Q}\rangle$ and $\langle\hat{Q}_{\text{FM}}\rangle$ quantitatively differ. This discrepancy originates from the fact that these operators are not diagonal in the measurement basis. Hence, in the experiments, an additional protocol is conducted to rotate the state to the natural basis of these operators. This second quench uses modified frequencies $\tilde{\Delta} = 0$ and $\tilde{\Omega} \neq \Omega_0$ to change the blockade radius of the Hamiltonian. In such a setting, the previously considered noise channels have modified rates while additional decoherence channels might appear. Furthermore, as the blockade radius decreases, states containing violations of the original constraint, such as vertices with double dimers, might be altered. Since these effects are absent from our rather predictive simulations, one can quantify the significant additional error induced when evaluating experimentally off-diagonal observables.

S4 Lattice topology and finite size effects

As discussed in the main text, when studying and describing topological states, the geometry and topology of the lattice (size, boundary conditions, genus) are of prime importance. The great flexibility of our method, unlike previous state-of-the-art techniques, enables us to directly investigate the dependence of the topological features of the system, namely the topological entanglement entropy, on its geometry and topology. To avoid finite-size effects, we consider larger lattices than in the main text with $N = 288$ atoms. By doing so, we are able to consider a lattice with open boundary conditions (similar to the one presented in Fig. 1) and a hole in the middle acting as an effective boundary, while the system still has a bulk to estimate the TEE. We first observe in Extended Data Fig. 5 that while the system is larger than that of the main text and topologically inequivalent, one observes a comparable value for the TEE $\gamma = 0.463(3)$, not closer to $\ln(2)$.

In order to conclude on finite-size effects, we additionally consider a smaller lattice of $N = 72$, with same boundary conditions. We observe a qualitatively similar behavior for the three topologically equivalent planar lattices. Despite always exhibiting a finite value of TEE, the value of γ for the smaller system is generally reduced, only attaining $\gamma = 0.435(4)$ at its peak. As shown in the main text, a susceptibility of γ to finite-size effects is only expected in the trivial phase. Furthermore, the simulation of a larger lattice than in the main text does not improve the topological entanglement entropy, reaching only $\gamma = 0.472(3)$. This further discards finite-size effects as the root for the departure from the characteristic γ value of $\mathbb{Z}_2$ order.

For a perfect QSL, the boundary conditions and the genus of the lattice only affect the degeneracy of the ground state of the system, having no impact on the value of the TEE. We verify this in Extended Data Fig. 5, where no qualitative difference is found between the topological entanglement entropy of the two lattices with same boundary conditions but different genus (0 for the planar lattice and 1 for the one with a hole). However, lattices with periodic boundary conditions reach slightly lower γ values, whereas systems defined on a cylinder possess higher γ than the ones on a torus.

Since the Kitaev-Preskill prescription yields correct results only if the correlation length is smaller than the domains defined for the extrapolation of the TEE, we decide to increase the size of the tripartition considered in order to verify the validity of our results. However, due to the size, shape and boundaries of the lattices considered, only two different tripartitions can fully fit within the bulk without any contact with the boundaries, as required. Therefore, we show in Extended Data Fig. 5**d** the TEE obtained with two different sizes of tripartition. In the case of the planar lattice, the effect seems to mainly be shifting the peak of γ to larger times, still reaching a similar value. For the toric lattice, the TEE does not reach the same peak as for the smaller tripartition, which might be due to the same shifting phenomenon, however stronger in this case. For both systems, we observed that the error on the numerical estimate increases with the size of the tripartition, reaching unphysical values for partitions touching the boundaries. In the case of a correlation length larger than the size of the domains, the value of γ should have reached larger values for the larger tripartition, which is not the case here. Therefore, we conclude that the smaller tripartition, as introduced in the main text, is the most appropriate for the calculation of γ, as it is large enough compared to the correlation length and it involves the lowest statistical error. For all tripartitions in the bulk considered, the TEE obtained numerically for an ideal RVB state matches $\gamma = \ln(2)$ within statistical error for either choice of boundary conditions.

References

[1] G. Semeghini, H. Levine, A. Keesling, S. Ebadi, T. T. Wang, D. Bluvstein, R. Verresen, H. Pichler, M. Kalinowski, R. Samajdar, A. Omran, S. Sachdev, A. Vishwanath, M. Greiner, V. Vuletić, and M. D. Lukin. Probing topological spin liquids on a programmable quantum simulator. *Science*, 374(6572):1242–1247, December 2021.

[2] Ruben Verresen, Mikhail D. Lukin, and Ashvin Vishwanath. Prediction of Toric Code Topological Order from Rydberg Blockade. *Physical Review X*, 11(3):031005, July 2021.

[3] Heinz-Peter Breuer and Francesco Petruccione. The Theory of Open Quantum Systems. Oxford University PressOxford, January 2007.

[4] J. R. Johansson, P. D. Nation, and Franco Nori. QuTiP: An open-source Python framework for the dynamics of open quantum systems. *Computer Physics Communications*, 183(8):1760–1772, August 2012.

[5] Andrew J. Daley. Quantum trajectories and open many-body quantum systems. *Advances in Physics*, 63(2):77–149, March 2014.

[6] Filippo Vicentini, Fabrizio Minganti, Alberto Biella, Giuliano Orso, and Cristiano Ciuti. Optimal stochastic unraveling of disordered open quantum systems: Application to driven-dissipative photonic lattices. *Physical Review A*, 99(3):032115, March 2019.

[7] Sepehr Ebadi, Tout T. Wang, Harry Levine, Alexander Keesling, Giulia Semeghini, Ahmed Omran, Dolev Bluvstein, Rhine Samajdar, Hannes Pichler, Wen Wei Ho, Soonwon Choi, Subir Sachdev, Markus Greiner, Vladan Vuletić, and Mikhail D. Lukin. Quantum phases of matter on a 256-atom programmable quantum simulator. *Nature*, 595(7866):227–232, July 2021.

[8] Yiyi Cai, Yu Tong, and John Preskill. Stochastic error cancellation in analog quantum simulation, November 2023.

[9] Chahan M. Kropf, Clemens Gneiting, and Andreas Buchleitner. Effective Dynamics of Disordered Quantum Systems. *Physical Review X*, 6(3):031023, August 2016.
